# Enabling liver transplantation during the COVID-19 era: More than screening donors and recipients for SARS-CoV-2

**DOI:** 10.6061/clinics/2020/e2377

**Published:** 2020-11-02

**Authors:** Edson Abdala, Daniel Reis Waisberg, Luciana Bertocco Haddad, Liliana Ducatti, Vinicius Rocha-Santos, Rodrigo Bronze de Martino, Wellington Andraus, Luiz Augusto Carneiro-D’Albuquerque

**Affiliations:** I Departamento de Gastroenterologia, Divisao de Transplantes de Figado e Orgaos do Aparelho Digestivo, Hospital das Clinicas (HCFMUSP), Faculdade de Medicina, Universidade de Sao Paulo, SP, BR; II Laboratorio de Investigacao Medica (LIM-37), Faculdade de Medicina (FMUSP), Universidade de Sao Paulo, SP, BR; III Departamento de Molestias Infecciosas e Parasitarias, Hospital das Clinicas (HCFMUSP), Faculdade de Medicina, Universidade de Sao Paulo, SP, BR

The consequences of severe acute respiratory syndrome coronavirus-2 (SARS-CoV-2) infection in the early postoperative course after liver transplantation (LT) are still not fully known. While data is scarce, it indicates that the incidence of early post-transplant coronavirus disease-19 (COVID-19) may reach up to 38% (1). Some centers have developed strategies for performing LT during the pandemic and, after implementing a multimodal stepwise approach, these facilities could minimize the risk of recipient SARS-CoV-2 infection (2,3). These measures may be summarized as establishing physically separated hospital facilities and in-hospital barrier protocols, performing rapid donor and recipient screening for SARS-CoV-2 once the organ becomes available, and optimizing recipient selection (2,3). We would like to share our experience, in which we initially observed a high post-LT COVID-19 infection rate in the first month of the pandemic, however we were able to reduce it significantly by adopting a similar approach, most notably by intensifying and expanding our barrier protocols.

Our institution, the Clinics Hospital of the University of São Paulo Medical School (HCFMUSP), is a medical complex located in São Paulo, Brazil, a city severely affected by SARS-CoV-2, with 160,337 confirmed cases documented by 1 July 2020. It is a public quaternary hospital and a major transplant center in Latin America, and it became the main referral center for severe COVID-19 cases when a city-wide quarantine was declared on March 24^th^. To maintain LT activity, a building specifically dedicated to non-COVID-19 patients was established. Second, we developed rapid screening protocols for donors and recipients, including epidemiological and clinical evaluation, real-time polymerase chain–reaction (RT-PCR) for SARS-CoV-2 from respiratory secretions, and chest computed tomography scans. Third, we tried to avoid using expanded-criteria donors and aimed to transplant more critical cases (*i.e.* patients with down-staged hepatocellular carcinoma or those with high model of end-stage liver disease scores, but with expected lower intensive care unit (ICU) stay), and fulminant hepatic failure cases. Finally, patients on the waiting list were fully informed about the risks of transplantation during the pandemic and we emphasized the importance of self-isolation afterwards.

In a first phase, which lasted from March 24^th^ to April 30^th^, we performed 14 deceased-donor liver transplantations (DDLT). Despite all of the adopted preventative measures, six recipients developed COVID-19 in the early postoperative period. One patient acquired SARS-CoV-2 in the community, and the other five acquired the disease during their index hospitalization, most probably secondary to nosocomial spread (4). We hypothesize that despite the screening of donors and recipients, our in-hospital barrier measures were not optimal, perhaps because our building was originally dedicated to the treatment of non-COVID-19 patients patients.

After this period, protocols with specific precautionary measures were reinforced and broadened, and we considered all patients and healthcare professionals as potential SARS-CoV-2 asymptomatic carriers. These protocols encompassed the operation rooms, ICUs, and hospital wards. We also established an exclusive ICU for transplanted patients. All hospital personnel were trained to wear personal protective equipment, including N95 masks, protective glasses, and gowns during all clinical activities. All patients were requested to wear surgical masks while within the hospital facilities. Moreover, hospital visitation was banned and patients were advised to maintain strict social isolation after discharge. At the same time, the government health agency also advocated universal mask use in the community. We limited transplantation procedures to extremely urgent cases for 2 weeks. As a result, in a second phase lasting from May 14^th^ to July 1^st^, we performed 14 DDLT and only one recipient developed COVID-19, most likely a community acquired infection (Table 1).

In conclusion, even though SARS-CoV-2 infection in the early postoperative period after LT has been reported by some centers (1,5-8), those that adopted multimodal strategies were able to successfully prevent such infections (2,3). By adopting more extensive in-hospital measures and by establishing tailored discharge plans, we reduced our infection rate from 42.85% to 7.14%, despite an increase in the number of new cases in the city. Since new waves of COVID-19 are expected in the future, we hope that this data may aid other centers in managing transplantation activities during the COVID-19 era.

## AUTHOR CONTRIBUTIONS

Abdala E, Waisberg DR and Haddad LB contributed in Conceptualization and Writing-Original Draft. Ducatti L, Rocha-Santos V and Martino RB contributed in Data curation, Writing-Review & Editing. Andraus W and Carneiro-D’Albuquerque LA contributed in Writing-Review & Editing and Supervision.

## Figures and Tables

**Table 1:** A summary of patients who received liver transplants at our facility during the COVID-19 pandemic.

	Phase 1	Phase 2
Time period	March 24^th^-April 30^th^	May 14^th^-July 1^st^
Number of DDLT	14	14
Early postoperative COVID-19 infection	6 (42.85%)	1 (7.14%)
Median recipienťs age	59.5 (41.25-64)	53.5 (40.5-69.25)
Recipienťs gender, male	8 (57.15%)	10 (71.43%)
ALF	1 (7.14%)	1 (7.14%)
HCC	5 (35.71%)	7 (50%)
Median MELD-Na	13 (11-21.5)	14 (10-20)
Pre-LT status		
Ward	13 (92.85%)	13 (92.85%)
ICU*	1 (7.14%)	1 (7.14%)
Median ICU stay (days)	4 (2-9)	3 (2-5)
Median hospital stay (days)	11 (7.5-25.5)	8 (7-10.25)
Mortality	5 (35.71%)	1 (7.14%)
Deaths due to COVID-19	2	0

Note: Continuous variables are presented as median and interquartile range (25%-75%).

* Both patients in the ICU were those transplanted due to ALF.

Abbreviations: ALF, acute liver failure; COVID-19, coronavirus disease 2019; DDLT, deceased-donor liver transplantation; HCC, hepatocellular carcinoma; ICU, intensive care unit; LT, liver transplantation; MELD, model of end-stage liver disease.
